# Cold saline irrigation of the renal pelvis during Radiofrequency Ablation of a central renal neoplasm: a case report

**DOI:** 10.1186/1752-1947-2-40

**Published:** 2008-02-07

**Authors:** Jose P Morales, Tarun Sabharwal, Marios Georganas, Renato Dourado, Declan Cahill, Andreas Adam

**Affiliations:** 1 Department of Interventional Radiology, Guy's and St Thomas' Foundation Hospital NHS Trust, London, UK; 2 Department of Urology, Guy's and St Thomas' Foundation Hospital NHS Trust, London, UK

## Abstract

**Introduction:**

Thermal destruction mediated by radiofrequency ablation (RFA) is gaining attention as an alternative treatment for patients with renal cell carcinoma (RCC), particularly in those who are not candidates for open surgery. Treatment of central tumours is occasionally associated with complications such as ureteric stricture, injury to the psoas muscle, haematuria and vascular laceration.

**Case presentation:**

We have used infusion of cold saline during RFA, through a retrograde ureteric catheter with its tip in the renal pelvis, in a patient with a central renal tumour.

**Conclusion:**

We believe this process to have successfully avoided the risk of thermal injury.

## Introduction

Thermal destruction mediated by radiofrequency ablation (RFA) is gaining attention as an alternative treatment for patients with renal cell carcinoma (RCC), particularly in those who are not candidates for open surgery [1]. RFA carries less risk in the treatment of peripheral RCC. Treatment of centrally located tumours is occasionally associated with complications such as ureteric stricture, injury to the psoas muscle, haematuria and vascular laceration [2]. However, protective measures can be taken to increase the safety of RFA when the electrode is located near the hilum of the kidney. We have used infusion of cold saline during RFA, through a retrograde ureteric catheter with its tip in the renal pelvis, in a patient with a central renal tumour in order to reduce the risk of thermal injury.

## Case presentation

**Figure 1:**
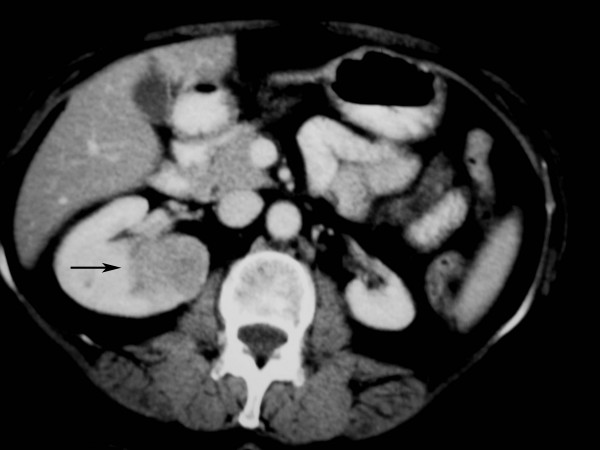
Contrast enhanced CT scan shows a right kidney mass suggestive of primary RCC measuring 3.2 × 3.2 × 3.8 cm with a central portion adjacent to the renal pelvis (black arrow).

A 62-year-old woman who had an incidental finding on computed tomography (CT) of a right renal mass was referred to our unit for RFA. The CT appearance of the tumor, which 3.2 × 3.2 × 3.8 cm, were consistent with (but not diagnostic of) primary RCC. Its medial margin was immediately adjacent to the renal pelvis (Fig 1). At the time of diagnosis the left kidney was atrophic. There was no evidence of metastases. The patient had been previously evaluated by a urologist expert in partial nephrectomy. However, given the size and location of the mass, and the fact that the contra-lateral kidney was atrophic, surgery was considered inappropriate following discussion at two consecutive multidisciplinary meetings at the referring hospital. The alternative of RFA with simultaneous cold saline irrigation of the renal pelvis was discussed at multidisciplinary meetings at both the referring hospital and our own institution and informed consent was obtained from the patient.

**Figure 2:**
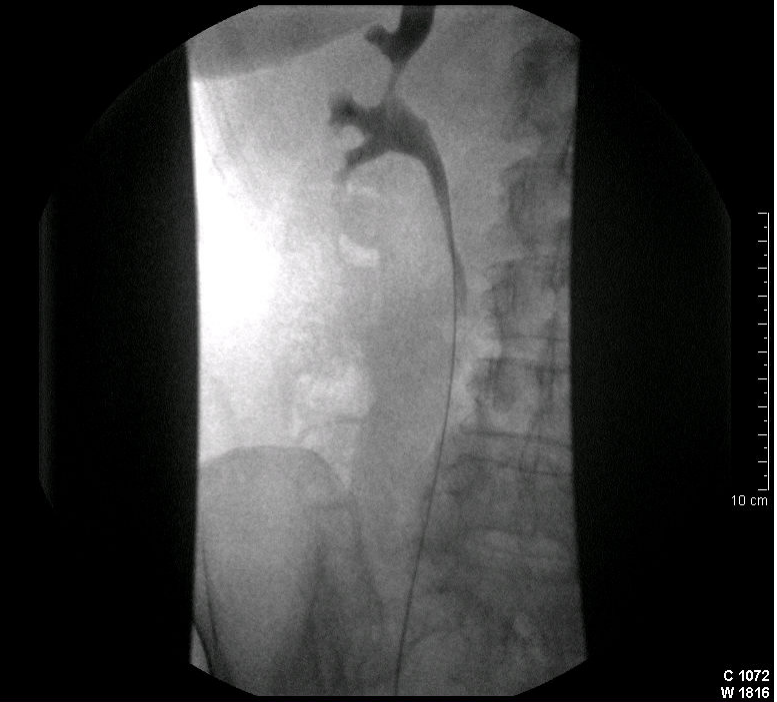
Contrast study shows ureteric catheter in-situ with the distal tip in the right renal pelvis.

**Figure 3:**
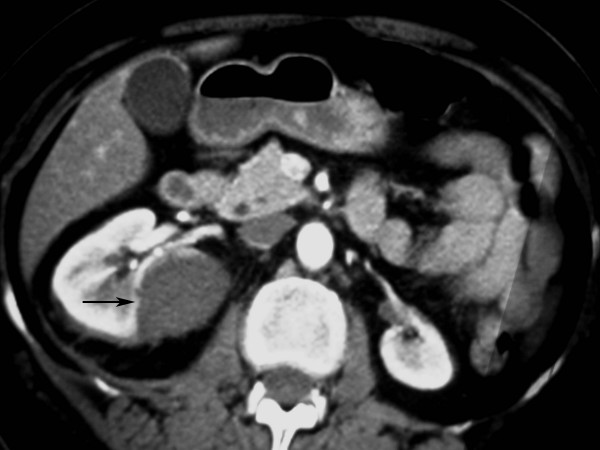
Follow-up CT scan at 24 hours showed the RF ablated area with no stricture of the renal pelvis and necrosis of tumour.

Prior to the RFA procedure, a 4.8 F retrograde ureteric catheter was inserted under endoscopic guidance and its tip advanced to the right renal pelvis (Fig 2). A Foley 14 F urethral catheter was inserted in the bladder and attached to a drainage bag. This system was used to irrigate the renal pelvis with cold saline (0.9%) at a rate of 1 l/hour. The irrigation was started 60 minutes before the procedure with saline at normal temperature and continued during the procedure with ice-cold saline. Subsequently the patient was transferred to the CT suite and unenhanced computed tomography images of the tumor were obtained. Following intravenous sedation and analgesia with 6 mg midazolam and 100 μg fentanyl, and infiltration of the percutaneous track with local anaesthetic (lidocaine 1%), a 20 G cutting needle was used to obtain a biopsy. Subsequently, a single 10 cm radiofrequency electrode (CoolTip, Radionics, Burlingthon, Mass) was placed into the tumor and used to ablate two adjacent areas for 12 minutes each. The temperatures reached 79°C and 82°C. The minimum distance of the electrode from the renal pelvis was 16 mm and 14 mm respectively. The total amount of normal and low temperature saline used during this procedure was 2.3 litres. The fluid emerging from the bladder catheter remained clear throughout the procedure. The ureteric and the Foley catheters were removed 30 minutes after the end of the procedure. A CT examination 24 hours later showed coagulation of the tumor and no evidence of hydronephrosis (Fig 3). The renal biopsy result demonstrated an oncocytoma. Follow-up CT at six months shows no evidence of recurrent tumour or hydronephrosis.

## Discussion

Asymptomatic renal tumours are being demonstrated with increasing frequency [3,4]. Although partial nephrectomy is the standard therapy for small RCC [2], minimally invasive options associated with limited morbidity–such as probe-ablative procedures–are, however, being investigated in selected patients for whom invasive, nephron-sparing surgery (whether laparoscopic or open) is undesirable. The main probe-ablative techniques being investigated as alternatives to partial nephrectomy are cryoablation, radiofrequency ablation, and high-intensity focused ultrasound. Advances in imaging, ablative system technologies, and early evidence that in situ tumor ablation can yield comparable results to those achieved with tumor resection in selected cases, have sparked significant interest in these minimally invasive techniques.

RFA is an increasingly employed alternative treatment option, especially in patients with significant co-morbidities or those who refuse surgery [1]. RFA of peripheral renal tumours is effective in achieving coagulation in most cases, because the kidneys are surrounded by fat, which prevents heat loss from the area being treated. In the case of central tumours, well-perfused tissue, vessels and the fluid-filled collecting system are found near the treatment area, potentially diminishing effectiveness and increasing the risk of complications. Ahrar et al. [5] reported four major complications, three of them in patients with solitary kidneys and central renal tumors. Additional procedures were necessary in these patients, such as insertion of a ureteric stent to deal with obstruction of the renal system by blood clot. Other complications associated with ablation of central renal tumors include injury, to the lumbar plexus, ureteral stricture, injury to psoas muscle and vascular laceration.

The treatment of renal lesions is complicated by heat loss, both conductive and convective. The kidney has a high blood flow, leading to substantial convective heat loss during renal RFA [2]. Some authors have managed to increase ablation diameters by reducing blood flow to the treated area using embolization or clamping of the renal artery [6]. The use and efficacy of generators of 200 W or greater and careful technique is helpful in achieving a satisfactory result [2].

As a new modality treatment of renal tumors, contra-indications to renal RFA are in evolution and are related with tumor in immediate proximity to a hollow viscus, renal pelvis or ureter. These contra-indications can be attempted to be reduced by the use of simultaneous irrigation with cold saline via ureteric catheter into the renal pelvis prior and during the ablation and in doing so, major complications may be avoided. This in keeping with previous reports were cooling systems were used to avoid complications of adjacent tissues in different organs. Dominique et al. [7] reported the feasibility of treating liver tumours close to a main biliary duct using intraductal cooling during RFA avoiding heat-induce damage and stenosis of biliary ducts. Similarly, Hiraki et al.[8]. published the use of RFA of metastatic mediastinal lymph nodes during cooling and temperature monitoring of the tracheal mucosa to prevent thermal damage. RFA with retrograde saline irrigation of centrally located renal parenchymal lesions had been proved safe and efficient in animal studies by Margulis et al.[9], which concluded that retrograde renal cooling helps protect the renal collecting system from injury during RFA without a decrease in expected lesion size. They also suggested that clinically retrograde renal cooling may decrease the risk of collecting system injury and subsequent complications during RFA. RFA with cooling system had been now reported in humans with the use of either normal saline or dextrose without significant differences in between them. [10,11]

## Conclusion

Although in our patient the biopsy revealed an Oncocytoma, it does not make any difference regarding the procedure itself as the lesion was close to the renal pelvis. We believe that using the ureteral irrigation catheter system we were able to prevent heat damage, either gross haematuria or ureteral stricture, as well as it may improve the impedance guarantying better ablation. This technique might be considered while performing RFA of central renal masses in order to avoid complications. It will be important for further evaluation of this technique in large clinical case series.

## Editor's note

Peer review was divided on the merits of this manuscript, and importantly also on the safety of using cold Saline rather than Dextrose. This is an area of clinical controversy and we urge readers to remember that this is only a single case report and that clinical decision making should always be based on the best available evidence.

## Competing interests

The author(s) declare that they have no competing interests.

## Authors' contributions

**JPM **carried out design and acquisition of data. Also was involved in drafting, editing and revising the manuscript critically for important intellectual content. **TS **was involved in revising the manuscript critically for important intellectual content and also had given final approval of the version to be published. **MG **carried out design and acquisition of data. Also was involved in drafting the manuscript. **RD **carried out design and acquisition of data. Also was involved in drafting the manuscript. **DC **was involved revising the manuscript critically for important intellectual content had given final approval of the version to be published. **AA **was involved revising the manuscript critically for important intellectual content had given final approval of the version to be published.

## Consent

Written informed consent was obtained from the patient for publication of this case report and any accompanying images. A copy of the written consent is available for review by the Editor-in-Chief of this journal.
